# Bridging patient-reported outcomes and performance assessments in older adults: linking the Short Physical Performance Battery to the standardised PROMIS Physical Function scale

**DOI:** 10.1093/ageing/afaf375

**Published:** 2026-01-28

**Authors:** Gregor Liegl, Audrey Yuki Brinker, Ursula Müller-Werdan, Andreas Heissel, Frank Buttgereit, Volker Köllner, Volkan Aykac, Udo Schneider, Felix H Fischer, Matthias Rose

**Affiliations:** Charité—Universitätsmedizin Berlin, corporate member of Freie Universität Berlin and Humboldt-Universität zu Berlin, Center for Patient-Centered Outcomes Research, Department of Psychosomatic Medicine, Berlin, BE, Germany; Charité—Universitätsmedizin Berlin, corporate member of Freie Universität Berlin and Humboldt-Universität zu Berlin, Center for Patient-Centered Outcomes Research, Department of Psychosomatic Medicine, Berlin, BE, Germany; Charité—Universitätsmedizin Berlin, corporate member of Freie Universität Berlin and Humboldt-Universität zu Berlin, Department of Geriatrics and Medical Gerontology, Berlin, BE, Germany; Evangelisches Geriatriezentrum Berlin gGmbH, Berlin, BE, Germany; University of Potsdam, Social and Preventive Medicine, Department of Sports and Health Sciences, Intra Faculty Unit “Cognitive Sciences”, Faculty of Human Science and Faculty of Health Sciences Brandenburg, Research Area Services Research and e-Health, Potsdam, BB, Germany; Sport-Gesundheitspark Berlin e.V., Centre for Sports Medicine, Berlin, BE, Germany; Charite—Universitatsmedizin Berlin, corporate member of Freie Universität Berlin and Humboldt-Universität zu Berlin, Department of Rheumatology and Clinical Immunology, Berlin, BE, Germany; Deutsches Rheuma-Forschungszentrum Berlin, Berlin, BE, Germany; Rehabilitation Clinic Seehof, Department of Psychosomatic Medicine, Teltow, BB, Germany; Charité—Universitätsmedizin Berlin, corporate member of Freie Universität Berlin and Humboldt-Universität zu Berlin, Department of Geriatrics and Medical Gerontology, Berlin, BE, Germany; Evangelisches Geriatriezentrum Berlin gGmbH, Berlin, BE, Germany; Charite—Universitatsmedizin Berlin, corporate member of Freie Universität Berlin and Humboldt-Universität zu Berlin, Department of Rheumatology and Clinical Immunology, Berlin, BE, Germany; Charité—Universitätsmedizin Berlin, corporate member of Freie Universität Berlin and Humboldt-Universität zu Berlin, Center for Patient-Centered Outcomes Research, Department of Psychosomatic Medicine, Berlin, BE, Germany; Charité—Universitätsmedizin Berlin, corporate member of Freie Universität Berlin and Humboldt-Universität zu Berlin, Center for Patient-Centered Outcomes Research, Department of Psychosomatic Medicine, Berlin, BE, Germany

**Keywords:** physical function, PROMIS, SPPB, physical performance, patient-reported outcomes, older people

## Abstract

**Background:**

Assessment of physical function, a key outcome in geriatric research, relies on either patient-reported or performance-based assessments. While several patient-reported instruments have been successfully linked to the standardised Patient-Reported Outcomes Measurement Information System (PROMIS) Physical Function scale, commonly used performance-based tools, such as the Short Physical Performance Battery (SPPB), remain uncalibrated to this scale. This lack of standardisation limits interpretability, comparability, and integration of physical function data across instruments, studies, and clinical settings.

**Objective:**

To link SPPB scores to the PROMIS Physical Function T-score metric in older adults.

**Methods:**

This analysis is part of the *Standardizing-PF* project, a prospectively designed cross-sectional study examining the possibility of mapping patient-reported and performance-based assessments onto a common scale. In the present study, 556 older adults (mean age 74 years) from different clinical and community-based settings subsequently completed a generic 20-item PROMIS Physical Function short form (PROMIS-PF20a) and the SPPB. Assumptions of item response theory modelling were investigated. We estimated a unidimensional item response theory-based linking model and derived cross-walks to convert SPPB scores into standardised PROMIS PF T-scores.

**Results:**

SPPB and PROMIS-PF20a were highly correlated (latent correlation = 0.89); assumptions of item response theory modelling were fulfilled. After linking, agreement between observed and linked T-scores was stable across several subsamples.

**Conclusions:**

The SPPB can be meaningfully linked to the PROMIS PF T-score metric, enabling standardised interpretation, comparison, and aggregation of performance-based and self-reported physical function in older adults. We provide a user-friendly score cross-walk table to facilitate application in clinical practise and standardisation in geriatric research.

## Key Points

- The SPPB can be validly linked to the standardised PROMIS PF T-score metric in older adults.
- Linking enables direct comparability between performance-based and self-reported functional assessments.
- SPPB score cut-offs of 3 (severe impairment), 9 (moderate impairment), and 11 (mild impairment) were derived based on PROMIS PF.
- We provide an easy-to-use score cross-walk table to enhance usability in both research and practise.
- The integration of performance-based and self-perceived function contributes to a comprehensive geriatric assessment.

## Introduction

With population ageing accelerating globally, maintaining physical independence has emerged as an essential goal in patient-centred care [1]. Physical function is a key outcome domain in ageing research and an important predictor of future disability, hospitalisation, and mortality in older adults [2, 3]. Accurately measuring physical functioning is therefore essential [4].

According to the Patient-Reported Outcomes Measurement Information System (PROMIS), physical function can be broadly defined as the ability to perform activities that require physical actions, including mobility and activities of daily living [5]. However, physical function is typically assessed using two fundamentally different types of clinical outcome assessment tools: patient-reported outcome (PRO) measures, such as questionnaires capturing self-perceived abilities; and performance outcome (PerfO) assessments, which are based on direct observation of standardised task execution [6]. PROs, based on self-report, provide insight into a person’s lived experience and are easy to administer [7]. PerfOs, on the other hand, generate more objective data but require greater resources and may be less representative of everyday functioning [8]. One prominent example for a widely used PerfO tool in geriatric settings is the Short Physical Performance Battery (SPPB), which is frequently applied to evaluate functional capacity in older individuals [9, 10].

Despite their complementary strengths and limitations, PRO and PerfO measures are not directly comparable, which poses significant challenges. First, there are no clear recommendations on which assessment tool to use [11]. As a result, different studies rely on different assessment types, making it difficult to compare, interpret, and aggregate findings, e.g. in systematic reviews or meta-analysis. For example, the association between physical function and mortality may vary depending on whether function was measured via PRO or PerfO, when assessment type-specific metrics are used [3]. Second, while current recommendations increasingly suggest the combined use of PRO and PerfO measures, e.g. for the comprehensive assessment of frailty [11, 12], true integration remains a methodological challenge. Without a standardised, assessment type-independent scale, combining these tools is inherently problematic—akin to comparing apples and oranges.

A promising solution is to use item response theory (IRT) modelling to link scores from established tools to standardised reference scales [13]. This psychometric approach has already proved its value in the context of PROs. For example, frequently used self-reported physical function scales, including geriatric-specific measures, have recently been calibrated onto the PROMIS Physical Function (PROMIS PF) T-score metric, enabling valid score comparisons across instruments [14–16]. Importantly, such standardised metrics enable the development of tailored instruments for specific populations—such as older adults—by selecting the most relevant items for that group, while still allowing direct score comparisons with other instruments calibrated to the same scale [5, 17].

Until recently, it was assumed that such linking might not be feasible across assessment types, since low correlations (e.g. Pearson’s r < 0.7) between PROs and PerfOs suggested distinct underlying constructs [18, 19]. However, much of this evidence stems from retrospective analyses of instruments that differ not only in assessment type, but also in domain coverage—e.g. using a very specific performance task (e.g. 6-minute walking test) compared with a broad PRO measure including various physical activities [20]. When PROs and PerfOs measure similar and more generic content, such as mobility or general functional capacity, emerging evidence suggests that the correlation is substantially higher, and meaningful linking becomes feasible [21]. In fact, recent findings suggest that the PROMIS PF metric can serve as a viable reference for linking selected PerfO batteries [22, 23]. Conversely, the SPPB has been shown its potential to be calibrated on a common scale with generic PRO items in older adults [24].

Against this background, we conducted the *Standardizing-PF* project, the first prospectively planned study investigating the feasibility of mapping PerfO- and PRO-based physical function measures to a standardised scale. This paper reports the results from linking the SPPB to the PROMIS PF metric in older individuals recruited from various clinical and community-based settings.

## Methods

### The *Standardizing-PF* project

This analysis is part of the *Standardizing-PF* project, a multicentre cross-sectional linking study designed to examine whether PRO measures and PerfO assessments of physical function can be mapped onto a common metric. The study was preregistered in the German Clinical Trials Register (DRKS00032125; registered 26 June 2023). The PROMIS PF metric was selected as the reference scale in this project, as it is standardised to the general population, includes clinically interpretable cut-off values, and allows comparisons across diverse clinical settings and language versions. Data were collected between December 2023 and February 2025 across different study sites in Germany (Charité—Universitätsmedizin Berlin, Reha-Zentrum Seehof, Evangelisches Geriatriezentrum Berlin, and Sport-Gesundheitspark Berlin), resulting in four subsamples: (i) geriatric inpatients, (ii) community-based older adults, (iii) rheumatology inpatients, and (iv) patients from an integrated psycho-cardiological rehabilitation programme for individuals with co-existing cardiac and mental disorders, which has recently been expanded to include post-COVID patients due to similar interactions of physical and psychological factors. Inclusion criteria included sufficient physical and cognitive capacity to participate in the assessments. Potentially eligible adult individuals were given written information about the study and asked to sign an informed consent form. All participants completed a subset of PROMIS PF items and subsequently underwent multiple PerfO tests, including the SPPB. The study is reported in accordance with STROBE guidelines [25] and funded by the German Research Foundation (DFG).

### Participants, sample size and handling of missing data in the present study

Because the SPPB was specifically developed to assess functional status in older adults [9], the present analysis included all participants from the geriatric and non-clinical older adult subsamples as well as participants aged 60 years and older from the rheumatology and rehabilitation subsamples. This resulted in a sample size well above the recommended requirement of N ≥ 500 considered sufficient for estimating accurate IRT model parameters [26]. To handle missing responses in PROMIS PF and SPPB items, we applied item-level imputation using the *mirt* package in R [27]. For this, a unidimensional graded response model (GRM) was freely estimated, and missing values were imputed based on the estimated model parameters to generate plausible values under the assumption of a single latent physical function trait.

### Measures

#### The PROMIS PF metric

The PROMIS PF item bank allows standardised assessment of self-reported physical function across various populations and captures a broad concept of functional ability [5]. The item bank is calibrated using IRT, specifically the GRM, enabling the use of item subsets to estimate individual scores on a common T-score scale. The PROMIS PF metric has a mean of 50 and a standard deviation of 10 in the general population; higher scores indicating better functioning. T-score cut-offs of 45, 40 and 30 have been suggested to indicate mild, moderate and severe functional impairment, respectively [28]. A key benefit of this IRT-based metric is that new items can be added—or instruments linked—without altering the meaning of the original scale [29]. Several commonly used instruments have already been successfully linked to the PROMIS PF metric, facilitating direct comparison across measures [14–16, 23].

In this study, we used the German PROMIS-PF20a short form, which is a widely used generic subset of the full item bank and provides good coverage of the broad PROMIS PF construct [30].

#### Short Physical Performance Battery

The SPPB is a widely used and psychometrically sound performance-based assessment tool specifically developed for evaluating functional capacity in older adults [9, 10]. It consists of three timed components: (i) a balance test (including side-by-side, semi-tandem, and full tandem stands), (ii) a gait speed test over a 4-metre walk, and (iii) a repeated chair stand test. Each component is scored on a 0–4 scale, resulting in a total score ranging from 0 to 12, with higher scores indicating better physical function. Sum scores ≤ 9 indicate functional impairment and is associated with all-cause mortality [31].

#### Additional measures

Participants also completed a general questionnaire that included sociodemographic variables and health-related information, including common medical problems as assessed with the German version of the Self-Administered Comorbidity Questionnaire [32], and a German single-item PROMIS pain intensity scale ranging from 0 (no pain) to 10 (worst imaginable pain), referencing the past seven days [33].

### Statistical analysis

The analyses were conducted in accordance with the PROMIS methodology and the linking procedures established in the PROsetta Stone project [13, 22, 34]. All analyses were conducted using R (version 4.3.1) [27, 35–39].

To describe the combined item set (PROMIS-PF20a and SPPB), we computed descriptive statistics, including corrected item-total correlations and internal consistency analysis. The latent correlation between PROMIS PF and the SPPB was examined using structural equation modelling. Before conducting the linking, key assumptions of unidimensional IRT modelling were tested [34]: monotonicity was assessed using Mokken analysis; unidimensionality was evaluated via confirmatory factor analysis (CFA) with a diagonally weighted least squares estimator and supplemented by a bifactor analysis to test for ‘essential’ unidimensionality. Measurement invariance across different subgroups was assessed via differential item functioning (DIF) analysis using ordinal logistic regression. DIF was tested for age (median split), gender (male/female), and pain intensity (median split), and medical problems (heart problems; rheumatological condition/arthrosis), which have previously been shown to be prone to non-invariance [5, 40]. Items were flagged if they showed statistically significant DIF with changes in pseudo-R^2^ ≥ 0.03 [5]. IRT item fit was evaluated using the generalised S-X^2^ statistic [34].

To link the SPPB to the PROMIS PF metric, we applied *fixed-item calibration* based on unidimensional IRT modelling [13] using the R package *mirt* [27]. Specifically, a GRM was fitted to the combined item set with PROMIS-PF20a items fixed to the originally established parameters [5], while SPPB item parameters as well as mean and variance of the latent trait distribution were freely estimated. With the resulting parameters, the SPPB was scored on the PROMIS PF metric using expected a posteriori (EAP) scores subsequently transformed to the T-score metric (‘IRT parameter linking’) [13]. From this model, we derived *cross-walk tables* that assign a single most likely PROMIS T-score to each possible SPPB sum score. These tables offer a user-friendly alternative allowing for straightforward score conversion without the need for statistical modelling, and have been shown to perform comparably to parameter-based scoring [41].

As a second approach, we conducted equipercentile linking, a non-parametric approach based on the empirical score distributions of SPPB and PROMIS PF [13]. This approach serves as a robustness check for potential violations of IRT assumptions; if results from both methods align, it supports the validity of the IRT-based linking approach. If not, equipercentile linking may be preferred, as this can indicate bias in the IRT-based estimates [13].

To evaluate the performance of the linked SPPB-based T-scores in comparison to the observed T-scores from the PROMIS-PF20a, we examined Pearson correlation, mean absolute error (MAE), root mean square error (RMSE), Bland–Altman plots, and standardised mean differences (SMDs) in predefined subsamples, with |SMD| values ≤ 0.2, ≤ 0.5, ≤ 0.8, and > 0.8 were interpreted as negligible, small, medium, and large effect sizes, respectively [41].

## Results

A total of 556 participants were included in the present analysis (65% female), the mean age was 74 years (SD = 9.2). For the pooled set of SPPB and PROMIS-PF20a items, the majority of participants provided complete data (*n* = 551; 99.1 %); no one had missing responses on all items.

On average, physical function was moderately impaired according to the PROMIS PF T-score metric (mean T = 38.4), with lowest scores found for the geriatric inpatient sample (mean T = 30.1, indicating severe impairment), and the highest scores for the non-clinical older adult sample (mean T = 47.2, indicating no relevant impairment). A similar pattern was observed for the SPPB, with an average sum score of 7.6 (indicating physical impairment). On average, mild to moderate pain intensity was reported. Detailed sample characteristics, including floor and ceiling proportions (i.e. the percentages scoring the minimum and maximum possible values) for both physical function measures, are provided in Table 1.

**Table 1:** Sample characteristics.

	Full sample N = 556 (100%)	Geriatric inpatients *n* = 226 (40.6%)	Non-clinical older adults *n* = 181 (32.6%)	Rheumatology inpatients *n* = 79 (14.2%)	Psycho- cardio rehab *n* = 70 (12.6%)
**Age in years; mean (SD)**	74.0 (9.2)	79.7 (7.4)	72.7 (8.0)	63.0 (3.6)	70.5 (7.9)
≤ 65 years; n (%)	135 (24.3)	7 (3.1)	42 (23.2)	25 (31.6)	61 (87.1)
66–75 years; n (%)	172 (30.9)	61 (27.0)	72 (39.8)	31 (39.2)	8 (11.4)
76–85 years; n (%)	183 (32.9)	108 (47.8)	53 (29.3)	21 (26.6)	1 (1.4)
86–95 years; n (%)	63 (11.3)	47 (20.8)	14 (7.7)	2 (2.5)	0 (0.0)
> 95 years; n (%)	3 (0.5)	3 (1.3)	0 (0.0)	0 (0.0)	0 (0.0)
**Female; n (%)**	358 (65.3)	138 (61.6)	133 (74.3)	47 (61.0)	40 (58.8)
**Comorbidities; n (%)**					
Heart problems	244 (45.4)	99 (45.6)	59 (33.3)	38 (48.7)	48 (72.7)
Hypertension	314 (57.7)	136 (61.8)	101 (56.1)	41 (52.6)	36 (54.5)
Lung problems	161 (30.3)	73 (33.5)	37 (21.1)	32 (41.6)	19 (30.6)
Diabetes	116 (21.8)	68 (31.1)	19 (10.8)	18 (24.0)	11 (17.7)
Gastrological problems	140 (26.4)	70 (32.4)	36 (20.3)	19 (25.3)	15 (23.8)
Kidney Diseases	94 (17.8)	56 (25.8)	16 (9.1)	20 (26.3)	2 (3.3)
Liver Diseases	27 (5.1)	13 (6.0)	5 (2.9)	6 (8.1)	3 (4.9)
Anaemia	68 (12.8)	34 (15.5)	10 (5.6)	21 (28.8)	3 (5.0)
Cancer	89 (16.8)	56 (25.8)	21 (11.8)	9 (12.0)	3 (4.9)
Depression	91 (17.2)	37 (17.1)	20 (11.4)	7 (9.2)	27 (43.5)
Arthrosis	260 (48.7)	103 (47.2)	98 (55.1)	33 (44.0)	26 (41.3)
Backpain	332 (62.5)	139 (63.5)	112 (62.9)	39 (53.4)	42 (68.9)
Rheumatological disease	95 (18.1)	24 (11.0)	9 (5.2)	60 (77.9)	2 (3.5)
**Pain intensity** ^a^ **; mean (SD)**	3.9 (2.4)	4.6 (2.4)	3.2 (2.1)	4.0 (2.5)	3.7 (2.2)
**PROMIS PF (PRO)** *					
Mean (SD)	38.4 (11.0)	30.1 (8.7)	47.2 (6.5)	37.1 (10.2)	43.5 (5.8)
Floor % / ceiling %	0.2 / 3.2	0.4 / 0.4	0.0 / 7.2	0.0 / 3.8	0.0 / 1.4
**SPPB (PerfO)** ^b^					
Mean (SD)	7.6 (4.3)	3.7 (3.3)	11.2 (1.1)	7.6 (3.3)	11.0 (1.7)
Floor % / ceiling %	10.4 / 27.2	24.3 / 1.3	0.0 / 57.5	3.8 / 10.1	0.0 / 51.4

*Abbreviations:* SD, standard deviation; PRO, patient-reported outcome; PerfO, performance outcome; PROMIS PF, Patient-Reported Outcomes Measurement Information System Physical Function; SPPB, Short Physical Performance Battery.

^a^Numeric rating scale ranging from 0 (no pain) to 10 (worst imaginable pain), referencing the past seven days.

^b^Sum score of the 3 SPPB items, ranging from 0 to 12; higher scores better functioning.

^*^T-scores with general population mean = 50, and standard deviation = 10; higher scores indicate better functioning.

Psychometric properties and results of assumption testing for IRT modelling are summarised in Table 2, along with evaluation thresholds based on established criteria [22, 34]. Internal consistency for the pooled item set was high (Alpha = 0.97, corrected item-total correlations ≥ 0.60). The association between PROMIS PF and SPPB was strong (latent correlation = 89). Mokken analysis indicated adequate scalability on scale and item level. Factor analysis supported sufficient unidimensionality, with a large portion of the common variance explained by one general factor (ECV = 0.73). DIF analysis indicated measurement invariance for all items across age, sex, pain intensity, and medical problems; unidimensional GRM calibration yielded appropriate item fit for all items.

**Table 2:** Psychometric properties of the pooled set of SPPB and PROMIS-PF20a items (combined scale of all 23 PRO and PerfO items).

Psychometric properties	Statistics / indices	Criterion	Results	
**Basic classic test theory statistics**				
Internal consistency	Cronbach’s Alpha	≥0.80	Alpha = 0.97	
Corrected item-total correlation	Correlation of an item with sum score of remaining items (r_itc_)	≥0.40	r_itc_ ≥ 0.60 for all items	
Association of SPPB with PROMIS-PF20a	Latent correlation^*^	≥0.75	Correlation = 0.89	
**Monotonicity**				
Mokken scale analysis	Scalability of the total scale (*H*)	≥0.50	*H* = 0.77	
	Scalability of individual items (*H_i_*)	≥0.30	*H_i_* ≥ 0.69 in all items	
	Graphical check whether ICCs are monotonically increasing		ICCs increasing in all items	
**Essential unimensionality**				
Exploratory bifactor analysis (three specific factors)	ECV *omegaH*	>0.60 >0.70	0.73 0.82	
	Salient general factor loadings	≥0.30	Loadings ≥0.57 in all items	
Confirmatory factor analysis (unidimensional)	CFI	≥0.95	*GRM*	*WLSMV*
			0.95	0.99
	RMSEA	≤0.08	0.07	0.09
	SRMR	≤0.08	0.04	0.04
	Sufficiently high standardised factor loadings	≥0.60	Loadings ≥ 0.83 in all items	
	Residual correlations of item pairs (r_Res_)	≤0.25	r_Res_ ≤ 0.14 in all item pairs	
**Differential item functioning**				
Age (median split)	Nagelkerke’s pseudo R^2^-change	≤3%	R^2^-change < 3% in all items	
Female vs. male			R^2^-change < 3% in all items	
Pain (median split)			R^2^-change < 3% in all items	
Heart problems vs. all others			R^2^-change < 3% in all items	
Rheumatological condition/arthrosis vs. all others			R^2^-change < 3% in all items	
**IRT model statistics**				
Unidimensional GRM fit	S-X^2^ p-value	≥0.001	*P* ≥ .016 in all items	

*Abbreviations:* CFI, comparative fit index, explained common variance; IRT, item response theory; GRM, graded response model; *H*, Loevinger’s Homogeneity coefficient; ICC, item characteristic curve; r_itc_, corrected item-total correlation; RMSEA, root mean square error of approximation; S-X^2^, generalised S-X^2^ item fit index; SRMR, standardised root mean square residual; TLI, Tucker-Lewis index; WLSMV, weighted least squares mean–variance adjusted.

^*^ *Correlation of the latent variables underlying the PPT and the PROMIS items, using CFA with two instrument-specific factors and a WLSMV estimator.*

**Figure 1:**
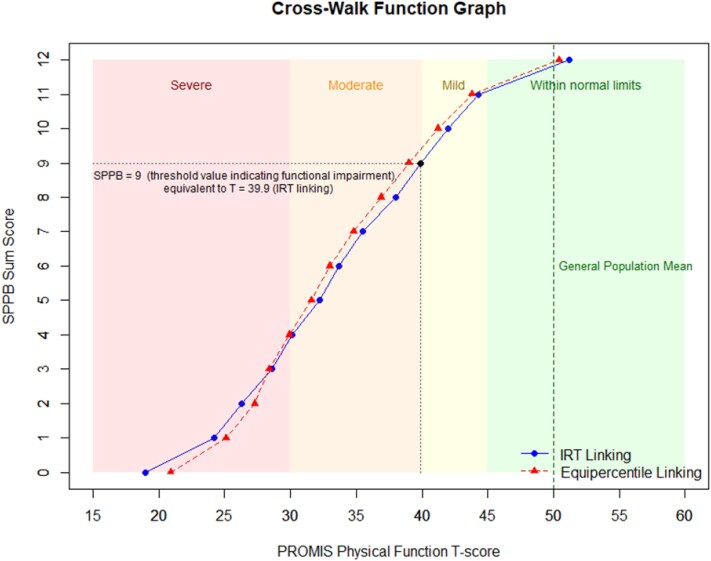
Score translation from SPPB to PROMIS Physical Function T-scores—cross-walk curves based on IRT-based (blue) and equipercentile (red) linking, illustrating the relationship between SPPB sum scores (y-axis) and PROMIS PF T-scores (x-axis). Coloured areas represent PROMIS severity ranges (red = severe impairment, orange = moderate impairment, yellow = mild impairment, green = within normal limits). An SPPB sum score of 9 (dashed lines) corresponds to a PROMIS T-score of ~40 across both linking approaches, marking the threshold for moderate functional impairment. The dashed green line indicates the general population mean (T = 50).

IRT-based and equipercentile linking yielded highly comparable score translations (Figure 1). In both approaches, an SPPB sum score of 9 (which is the commonly used threshold for indicating functional impairment) corresponded to a T-score of approximately 40, aligning with the established PROMIS cut-off for moderate impairment. The association between linked (SPPB-based) and observed (PROMIS-PF20a) T-scores was strong (r ≥ 0.80), and the level of agreement was similar across linking approaches (MAE ≤ 5.3, RMSE ≤ 6.8; see Table 3).

**Table 3:** Agreement of observed (PROMIS-PF20a) and linked (SPPB-based) T-scores.

Statistics	PROMIS-PF20a (observed T-scores)	SPPB (linked T-scores)		
		IRT parameter linking	IRT-based cross-walk linking	Equipercentile linking
T-score mean (SD)	38.4 (11.0)	38.4 (10.8)	38.4 (10.7)	38.1 (10.0)
T-score range: Min.; Max.	6.9; 60.6	19.0; 51.1	19.0; 51.1	20.8; 50.4
Pearson correlation with observed T-scores		0.81	0.80	0.80
MAE *(observed versus linked T-scores)*:		5.2	5.3	5.1
RMSE *(observed versus linked T-scores)*:		6.8	6.8	6.6
SMD [95% CI] *(observed–linked T-score)*:				
Full sample		0.00 [−0.08; 0.08]	0.00 [−0.09; 0.08]	0.03 [−0.05; 0.12]
Female		−0.11 [−0.21; 0.00]	−0.11 [−0.22; 0.01]	−0.08 [−0.18; 0.03]
Male		0.18 [0.04; 0.33]	0.17 [0.03; 0.31]	0.22 [0.07;0.36]
Age < median		−0.11 [−0.22; 0.01]	−0.11 [−0.23; 0.00]	−0.04 [−0.16; 0.07]
Age ≥ median		0.11 [−0.01; 0.23]	0.10 [−0.02; 0.22]	0.11 [−0.01; 0.23]
Geriatric inpatients		0.20 [0.07; 0.33]	0.17 [0.04; 0.30]	0.13 [0.00; 0.26]
Non-clinical older adults		−0.06 [−0.20; 0.09]	−0.03 [−0.18; 0.11]	0.09 [−0.06; 0.23]
Rheumatology		−0.06 [−0.28; 0.16]	−0.09 [−0.31; 0.14]	−0.01 [−0.23; 0.22]
Psycho-cardio rehab		−0.65 [−0.91; −0.39]	−0.63 [−0.89; −0.37]	−0.50 [−0.75; −0.25]

*Abbreviations:* CI, confidence interval; IRT, Item-Response Theory; Max., maximum; Min., minimum; PROMIS-PF20a, 20-item short form of the Patient-Reported Outcomes Measurement Information System Physical Function item bank; SMD, standardised mean difference for paired samples; SD, standard deviation.

Agreement between observed and linked scores was consistent across predefined subsamples, with all SMDs ≤ 0.2 (negligible effect size)—except for male participants in the equipercentile approach (small effect size), and participants from the (psycho-)cardiology rehabilitation group, where all linking methods showed negative medium effect sizes. This suggests that participants in this group showed worse physical function in the PROMIS-PF20a than would be expected based on SPPB scores. Bland–Altman plots confirmed high agreement across assessment types, with negligible site effects except in the psycho-cardiology group (Appendix Figures S1 and S2).

The score cross-walk for the IRT-based linking approach (recommended method) is shown in Table 4. Individual IRT-based item parameters for the SPPB items are provided in Appendix Table S1; freely estimated group parameters of the latent trait distribution are presented in Appendix Table S2.

**Table 4:** Crosswalk table between SPPB sum scores and PROMIS T-scores

SPPB sum score	PROMIS PF T-score	*SE*	PROMIS score cut-points	SPPB threshold for impairment	N
0	19.0	6.1	Severe Impairment	Functional impairment	58
1	24.2	4.9			31
2	26.3	4.8			22
3	28.6	4.6			18
4	30.1	4.9	Moderate impairment		20
5	32.2	4.6			22
6	33.7	4.6			32
7	35.6	4.5			24
8	38.8	4.9			31
9	40.0	4.8			31
10	42.0	4.9	Mild impairment	No functional impairment	47
11	44.3	4.9			69
12	51.1	6.8	Within normal limits		151

*Note:* Use the SPPB sum score to find the corresponding PROMIS T-score and *SE*, which indicates the precision of the score. Interpret T-scores based on suggested threshold categories.

*Abbreviations:* N, sample size for each score on the SPPB; PROMIS PF, Patient-Reported Outcomes Measurement Information System physical function metric; *SE*, standard error.

## Discussion

In this prospectively designed study, we linked the SPPB, a widely used performance-based measure of functional capacity, to the standardised PROMIS PF T-score metric in older individuals. We found that key assumptions of IRT-based linking were met for the SPPB and that linking yielded consistent results across methods and subsamples. This supports the suitability of mapping SPPB scores to the PROMIS PF scale, thus enabling direct comparability between performance-based and self-reported assessments of physical function.

In line with methodological recommendations [13], we prioritised the IRT-based approach for its conceptual strengths and similar empirical performance compared to equipercentile linking in our data. For routine application, we provide a practical cross-walk table, allowing users to convert SPPB sum scores into PROMIS PF T-scores without complex modelling [41]. At the same time, IRT parameter-based scoring remains possible and may be especially useful when individual SPPB items are missing or not assessable (e.g. due to space or safety constraints).

Linking the SPPB to the PROMIS PF metric offers multiple advantages. First, using the standardised T-score metric as a reference scale, which is normed to the general population [5], adds clinical meaning and standardised interpretability across assessment types. Based on our results, the following SPPB sum score cut-offs can be suggested: 0–3 as severe, 4–9 as moderate, and 10–11 as mild functional impairment (see Table 4). In this context, our finding that the conventional SPPB threshold for functional impairment (≤9) aligned with the PROMIS cut-off for moderate limitation (T = 40) supports the validity of the linking. Moreover, SPPB results can be interpreted in view of PROMIS PF reference scores with respect to country, age, and gender, e.g. using a simple web application (http://www.common-metrics.org/PROMIS_PF_and_PI_Reference_scores.php [42]). Second, the linking enables harmonised measurement across studies and facilitates synthesis of evidence in meta-analyses or health systems research. Legacy SPPB data can now be retrospectively translated into PROMIS T-scores, placing the SPPB on the same metric as other physical function instruments that have already been linked to PROMIS PF [14, 17, 22] and ensuring compatibility with future tools that will be mapped to the same scale [13]. Third, placing PRO and PerfO scores on a common metric enhances interoperability and supports both the use of one measure when the other is unavailable and the construction of composite scores [24], in line with emerging recommendations to combine PROs and PerfOs in geriatric assessment [12]. Fourth, the use of a common metric permits direct quantification of individual-level discrepancies between self-perceived and ‘objective’ functioning [23]. Such discrepancies may reflect clinically relevant factors and could reveal important insights into individual perceptions of health and functioning, and could serve as a relevant health predictor. For example, it has been shown that discrepancies between subjective and objective health predict the fall risk in older adults [43].

Notably, while we observed high agreement across most subsamples on the group-level, an exception emerged in patients from (psycho-)cardiology rehabilitation, where all linking approaches revealed discrepancies between observed and SPPB-derived T-scores—in the direction that participants rated their physical function lower than would be expected based on their actual performance. This likely reflects a systematic difference in response behaviour rather than a failure of the model. For example, existing literature suggests that individuals with fatigue may underestimate their functional ability in self-reports [23]. Importantly, in a sensitivity analysis (data not shown), exclusion of this subsample did not affect the IRT-based linking function, supporting the robustness of our results. Interestingly, further exploratory analysis indicated these discrepancies were mainly driven by the small subset of only 24 post-COVID participants (large effect size), while only small effect sizes were found for the remaining psycho-cardiology rehabilitation patients (*n* = 46). In this context testing of site-level DIF was not feasible due to sample-size restrictions; future studies with larger and more balanced groups should examine site-related invariance more closely. Until confirmed in larger samples, we recommend cautious use of the cross-walks in post-COVID and psycho-cardiological patients.

This study has further limitations. First, no external validation sample was available to independently confirm the cross-walk. Nevertheless, agreement between observed and linked T-scores was stable across several subsamples indicating validity of the established linking algorithm. Second, while the linking allows for reliable group comparisons across instruments, individual-level scores may differ considerably in some cases. However, this also applies to linking studies within a specific assessment type [41]. In this context, the SPPB provides only 13 discrete values (sum scores between 0 and 12), which results in relatively high ceiling proportions in some subsamples and limits precision at the individual level. However, standard errors (see Table 4) are comparable to linked PRO instruments with similarly limited numbers of response options (e.g. numeric rating scales) [15]. Finally, generalizability to other populations remains to be established. Future research should aim to replicate these findings in independent samples, examine performance in populations with post-COVID conditions or mental health comorbidities, and extend the linking approach to other performance-based functional assessments.

## Conclusion

In summary, this is the first study demonstrating that the SPPB can be validly linked to the PROMIS PF metric, providing a standardised approach for integrating performance-based and self-reported assessments of physical function. The resulting score cross-walk enables meaningful comparisons across instruments and supports harmonised outcome measurement in geriatric research, clinical assessment and care planning. These findings represent an important step toward standardised person-centred assessment in ageing populations.

## Supplementary Material

aa-25-2166-File002_afaf375

## Research Data Transparency and Availability

The data that support the findings of this study are available from the corresponding author upon reasonable request.
