# Supplementary material for: Bridging patient-reported outcomes and performance assessments in older adults: linking the Short Physical Performance Battery to the standardised PROMIS Physical Function scale
Source: Age Ageing. 2026 Jan 28;55(1):afaf375. doi: 10.1093/ageing/afaf375 (PMC12848933; doi:10.1093/ageing/afaf375)
Supplement: aa-25-2166-File002_afaf375 [file aa-25-2166-file002_afaf375.docx]

**Supplemental Appendix**

**Bridging Patient-Reported Outcomes and Performance Assessments in Older Adults: Linking the Short Physical Performance Battery to the Standardised PROMIS Physical Function Scale**

|  | **Page** |
| --- | --- |
| **Figure S1:** Bland–Altman plot showing agreement between observed (PROMIS-PF20a) and IRT-linked SPPB-based T-scores across the total sample. | 2 |
| **Figure S2:** Bland–Altman plot showing agreement between observed (PROMIS-PF20a) and equipercentile-linked SPPB-based T-scores across the total sample. | 3 |
| **Table S1:** SPPB item parameters and fit statistics after IRT-based linking to PROMIS PF with fixed PROMIS-PF20a item parameters | 4 |
| **Table S2:** Freely estimated group parameters of the latent trait distribution in the IRT-based fixed-item calibration model | 5 |


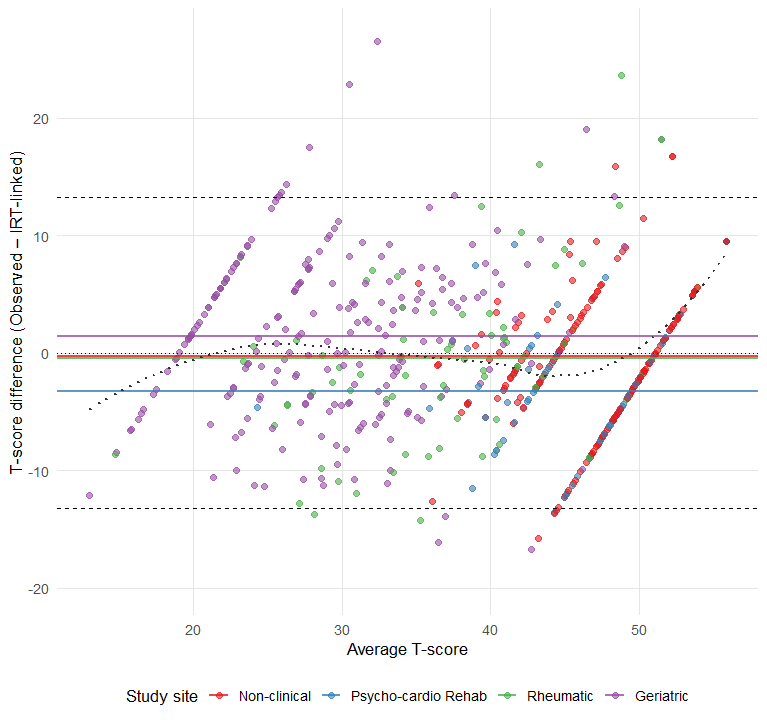


**Figure S1:** Bland–Altman plot showing agreement between observed (PROMIS-PF20a) and IRT-linked SPPB-based T-scores across the total sample. Dots represent individual participants, and colors indicate the different study sites. The dotted black line represents the overall mean difference along the T-score continuum, and dashed black lines show the 95 % limits of agreement. Solid colored lines indicate the mean T-score difference within each study site, highlighting potential site-specific deviations.


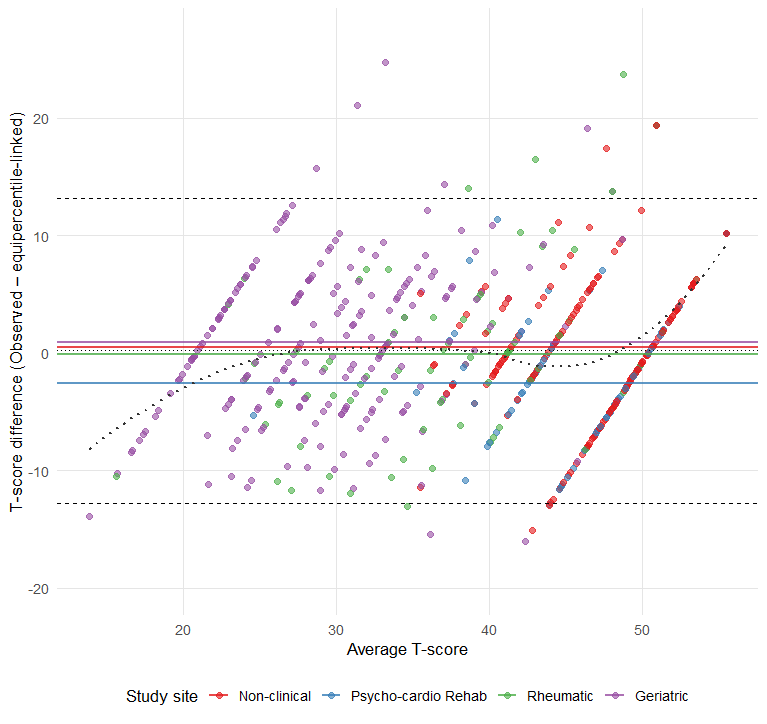


**Figure S2:** Bland–Altman plot showing agreement between observed (PROMIS-PF20a) and equipercentile-linked SPPB-based T-scores across the total sample. Dots represent individual participants, and colors indicate the different study sites. The dotted black line represents the overall mean difference along the T-score continuum, and dashed black lines show the 95 % limits of agreement. Solid colored lines indicate the mean T-score difference within each study site, highlighting potential site-specific deviations.

**Table S1:** SPPB item parameters and fit statistics after IRT-based linking to PROMIS PF with fixed PROMIS-PF20a item parameters

| **Item** | | **GRM fit** | **GRM item parameters [95% CI]** | | | | |
| --- | --- | --- | --- | --- | --- | --- | --- |
| **Item ID** | **Item description** | **S-X^2^**  **p-value** | **a** | **b1** | **b2** | **b3** | **b4** |
| SPPB_1 | Balance Tests | 0.295 | 2.017 | -2.489 | -2.167 | -1.671 | -1.521 |
| SPPB_2 | Gait Speed Test | 0.035 | 2.422 | -2.745 | -2.319 | -1.784 | -1.266 |
| SPPB_3 | Chair Stand Test | 0.295 | 2.546 | -1.706 | -1.277 | -0.899 | -0.446 |

*Abbreviations:* IRT, item response theory; GRM, graded response model; PROMIS-PF20a, 20-item short form of the Patient-Reported Outcomes Measurement Information System physical function item bank; SPPB, short physical performance battery; S-X^2^, generalized S-X^2^ item fit index

**Table S2:** Freely estimated group parameters of the latent trait distribution in the IRT-based fixed-item calibration model

| **Group Parameter** | **Estimate** |
| --- | --- |
| Mean | −1.17 |
| Variance | 1.21 |
